# The risk of pathogenic intestinal parasite infections in Kisii Municipality, Kenya

**DOI:** 10.1186/1471-2458-8-237

**Published:** 2008-07-14

**Authors:** Robert M Nyarango, Peninah A Aloo, Ephantus W Kabiru, Benson O Nyanchongi

**Affiliations:** 1 Department of Health Sciences, Gusii Institute of Science and Technology, P.O. Box 222-40200 Kisii, Kenya; 2 Department of Zoological Sciences, Kenyatta University P.O. Box 43844-01000 Nairobi, Kenya; 3 Department of Clinical Pathology, Kenyatta University P.O. Box 43844-0100 Nairobi, Kenya; 4 Department of Experimental Medicine and Public Health, Via Camillo Lili n. 55-62032 Camerino (MC), Italy

## Abstract

**Background:**

Intestinal parasitic infections are among the most common infections worldwide. Various epidemiological studies indicate that the prevalence of intestinal parasites is high especially in developing countries, although in many of these, the environmental risk factors have not been clearly elucidated. The objective of this study was to determine the risk of pathogenic intestinal parasites infections in Kisii Municipality.

**Methods:**

Random sampling was used in the selection of the study samples. Stool parasitological profiles of food handlers were done by direct smear and formalin-ethyl acetate sedimentation method. Both vegetable and meat samples were examined for the presence of intestinal parasites. The storage and meat handling practices of the various butcheries were observed.

**Results:**

Types of samples examined for occurrence of intestinal parasites includes, a total of 84 vegetable, 440 meat and 168 stool samples. Fifty five (65.5%) vegetable, 334 (75.9%) meat and 69 (41.1%) of the stool samples were found positive for intestinal parasites indicating a high overall risk (66.18%) for intestinal parasite infections. Of the parasites detected, the most common parasites infesting the foodstuffs and infecting the food handlers were *Ascaris lumbricoides *and *Entamoeba histolytica*. Parasites were significantly less likely to be present on meat that was refrigerated during display than meat that was displayed at ambient temperature.

**Conclusion:**

There is a high risk of infection with intestinal parasites in the sampled Municipal markets. About half of the food handlers surveyed (41.1 %) at the Municipal Hospital had one or more parasitic infections. Furthermore, meat (65.5%) and vegetables (75.9%) sold at the Municipal market were found to be contaminated with parasites hence the inhabitants requires a need for education on food safety, good distribution practices and improvement on sanitary conditions.

## Background

Intestinal parasites cause significant morbidity and mortality throughout the world, particularly in developing countries and in persons with comorbidities. The risk of intestinal parasites infections is measured using the DALY (disability- adjusted life year) and one DALY represents the loss of one year of equivalent full health [1]. The resulting diseases have socio-economic impact in terms of high treatment costs per DALY and hospitalization costs [2]. Globally, millions of people suffer from parasitic infections such as *Ascaris lumbricoides *(1.2 billion), *Trichuris trichiura *(795 million), hookworms (*Ancylostoma duodenale *and *Necator americanus*) (740 million) [3], *Entamoeba histolytica *(50 million) [4] and *Giardia lamblia *(2.8 million) [5]. In man, intestinal parasites are significantly associated with diarrhoea [6]. The fecal oral route is significant in the transmission of parasitic infections to humans via poor personal hygiene [7], environmental conditions like contamination of soil and water sources with human faeces [8] and poor sewage disposal such as use of night soil as fertilizer [9]. When the soil becomes contaminated, the eggs in soil can be transferred onto vegetables then onto the hands and transferred directly into the mouth [10] or ingested by eating raw vegetables [9]. The risk of intestinal parasites is even higher among the inhabitants of towns of the developing countries especially in the shanties and slums where there is poor disposal of garbage, poor health systems and overcrowding [11]. Intestinal parasites have been found to adhere to vegetables, fruits, fingers, utensils, door handles [12] and money [11]. Additionally, they can be transmitted through flies [13] and contaminated fingernails [14].

Epidemiological surveys done in Kenya's poor periurban and urban school children revealed a high prevalence of intestinal parasitic infections with *Ascaris lumbricoides *(82%), *Trichuris trichiura *(60%), *Entamoeba histolytica *(41%) and *Giardia lamblia *(30%) [15]. Recent studies done at various locations in Kisii Highlands, Western Kenya, showed that there was high prevalence of many intestinal parasites such as *A. lumbricoides *(10%), hookworm (4%) and *T. trichiura *(0.1%) [16]. The Kisii Municipal markets are characterized by a lack of bathrooms and washing facilities, poor sanitary conditions of latrines which lack water supplies and a frequent presence of piles of garbage that provide a fertile environment for transmission of intestinal parasites. The objective of this study was to determine the prevalence of intestinal parasites among food handlers and foodstuffs sold at Kisii Municipality.

## Methods

### Study area

The study was carried out between December 2004 and June 2005 in Kisii Municipality, which is located at the southern end of the western Kenya highlands at an altitude of 1660 m above sea level. The area receives average rainfall of over 1500 mm per annum distributed almost throughout the year although there are two rainy seasons (March to May and October to November). Temperatures range from 10°C to 30°C with relative humidity of 88%. The area is densely populated with a population of 37,531 people and a density of 1295 people/km^2 ^[17]. Majority of the population depends on open air markets as a source of both meat and vegetable foodstuffs although there is poor drainage and sanitation characterized by presence of refuse dump sites nearby. The parasitological survey of the food handlers and foodstuffs was carried out at the Kisii District Hospital.

### Study population

A correrational descriptive study design was used to determine the association between various risk factors and the occurrence of intestinal parasites. Two open air markets were randomly selected by lottery method from the list of the five markets of Kisii Municipality. The sample size for foodstuffs and food handlers was calculated on a prevalence of 10%, d = 0.05 at a confidence level of 95%. Each of the four varieties of vegetables commonly sold in the study area was selected by random sampling from the vendors in the open air markets. Samples of vegetables leaves weighing 250 g for each variety (kales (*Brassica oleracea var. acephala*), cabbage (*Brassica oleracea capitata*), spider flower (*Gynandropsis gynandra*) and black nightshade (*Solanum nigrum*)) were collected each day from the vendors by random sampling. The leaves of the four vegetable varieties were chosen because they are the major ones eaten by residents of Kisii Municipality. Twenty two butcheries were randomly selected using lottery method from using a list of the total of 125 butcheries in the Municipality. The asymptomatic adult male and female food handlers from the sampled markets and butcheries were randomly selected by lottery method from a list of attendance at Kisii District Hospital for routine examination of stool parasitological profile according to the Kenyan Ministry of Health Regulations (Public Health act Chapter 242) [18]. The meat (beef, mutton and goat) samples each weighing 250 g was collected from the 22 butcheries selected. The samples were collected weekly, between 9.00 a.m. and 12.00 mid day. The meat handling practices at the butcheries were observed on every sampling occasion using a checklist [see Additional file 1] and the observations recorded. However, this study did not examine parasitological profile of slaughter houses and hand washes of foodstuff vendors. Each of the food handler selected was given a plastic stool container and asked to bring bean-sized stool sample within 24 hours. This study was approved by the research and ethics committee of Kenyatta University as well as the Ministry of Science and Technology, Kenya. Consent of the study participants was obtained before taking the samples for stool examination. The food handlers alternate between the five markets in Kisii Municipality, although the two markets chosen in this study are the major ones. Double counting was avoided by asking the food handlers if they had previously been examined for intestinal parasite profile.

### Sample analysis

A 250 g sample of each vegetable or meat sample was examined for intestinal parasite profile (described by [12]). The sample was washed in distilled water and the suspension was strained through a sterile sieve to remove undesirable materials. The filtrate was centrifuged and supernatant discarded while the deposit was suspended in magnesium sulphate floatation fluid of specific gravity 1.3 and recentrifuged. The floatation fluid was filled to the brim and a cover slip was superimposed. The cover slip was lifted and examined under a light microscope. The cysts and eggs of various parasite species present were identified [19]. Each parasite eggs, larvae or cysts present in the samples were counted and densities of each species were expressed as "many" (>three cysts per high-power field; >20 eggs or larvae per mount); "moderate"(two cysts per high-power field; 10 to 19 eggs or larvae per mount); "few" (one cyst per high-power field; three to nine eggs or larvae per mount); and "rare" (two to five cysts and <two eggs or larvae per mount). For simplification, numerical values were assigned to each density: many, 4; moderate, 3; few, 2; rare, 1; and none, 0 [20]. The food handling practices of the butcheries including the meat storage method(s), the handlers' hygiene standards and the presence of houseflies on meat samples were observed using a check list and recorded during each sampling day. A direct saline smear preparation of the stool sample specimens obtained from the food handlers were made for examination of trophozoites, ova and cysts of intestinal parasites using Lugol's Iodine solution and formalin-ethyl acetate sedimentation method [21].

### Statistical analysis

A computer program (SPSS 11.5 for Windows) was used for data analysis. Multiple logistic regression analysis was used. The differences were considered to be statistically significant when the p-value obtained was less than 0.05

## Results

### Parasites in the foodstuffs

A total of eighty four vegetable samples comprising of 21 samples for each species were collected, kales cabbage, spider flower and black night shade. In all, 55 (65.5%) vegetable samples were infested with intestinal helminthes. Eleven (52.4%) of kales, 12 (57.1%) cabbages, 17 (81.0%) spider flower and 15 (71.4%) of black night shade tested positive. An average parasite score density of 2.37 was observed in the vegetables (Table 1). A statistically significant difference was observed between infestation rates of intestinal parasites and types of vegetables (p = 0.000)).

**Table 1:** The prevalence and density of intestinal parasites among vegetable foods samples.

Samples	n	Frequency of intestinal parasites (%)	Score of parasite density
Kales	21	11(52.4)	2.43
Cabbage	21	12(57.1)	1.86
Spider flower	21	17(8 1)	2.57
Black night shade	21	15(71.4)	2.62
Overall	84	55(65.5)	2.37

The risk factors (independent variables) influencing infection with intestinal parasites (dependent variable) were investigated in 22 butcheries (Table 2). These were the meat storage methods, presence or absence of cashier, the presence or absence of protective clothing and the presence or absence of houseflies on meat samples. The storage methods used included placing the meat in refrigerator 4 butcheries (18.2%), placing the meat on the surface of the table 10 (45.5%), and hanging the meat in a wire mesh 8 (36.4%). In the meat stored in a refrigerator, there were 15 (18.8%) samples with intestinal parasites, while those placed in the open surface had 73 (45.6%) samples and wire mesh placed had 73 (36.5%) samples with intestinal parasites. Parasites were significantly less likely to be present on meat that was refrigerated during display than meat that was displayed at ambient temperature (p = 0.002).

**Table 2:** The relationship between risk factors for meat handling practices and prevalence of intestinal parasites.

Risk factor	No of samples	Frequency of intestinal parasites (%)	Score of parasite density
Storage Method			
Refrigerator	80	31 (7.0)	1.40
Open surface	200	165 (37.5)	1.87
Wire mesh	160	138 (31.4)	1.63
Cashier			
Absent	300	263 (59.8)	1.85
Present	140	71(16.1)	1.64
Protective clothing			
Present	200	134(30.5)	1.50
Absent	240	200(45.5)	1.61
Houseflies			
Present	300	263(59.8)	1.83
Absent	140	71(16.1)	1.64

Fifteen (68.2%) butcheries had the same person handling the meat and the cash (no cashier) while seven (31.8%) had a food handler and a cashier. There was a significantly high prevalence of intestinal parasites in meat samples where there was no cashier 154 (59.2%) than where the cashier was present 74 (40.9%) (p = 0.000).

The prevalence of intestinal parasites in meat samples collected from (47.1%) whereas those present from butcheries where personnel were not wearing protective clothing were 233 (52.9%).

The numbers of butcheries where there were houseflies on the meat samples collected were 13 (59.1%) while those that did not have houseflies were 9 (40.9%). The average parasite score density in the meat samples was ranging from 1.40 to 1.87.

### Parasitological profiles of food handlers

Stool specimens were collected from the food handlers attending the Kisii District Hospital. Out of 168 food handlers, 69 (41.1%) were infected with one or more intestinal parasites of whom 27 (16.1%) were infected with one species of protozoan, 37 (22.0%) were infected with one species of helminth, 5(3.0%) had mixed infections, with 3 (1.8%) having *Entamoeba histolytica *and *Ascaris lumbricoides*, and 2 (1.2%) had *E. histolytica *and *Giardia lamblia *(Table 3). Of the most common intestinal parasite species were *A. lumbricoides *22 (13.1%) and *E. histolytica *20 (11.9%). The others were hookworms 13 (7.7%), *G. lamblia *6 (3.6%), *Trichuris trichiura *2 (1.2%) and *Balantidium coli *1(0.6%), in their decreasing order of prevalence. There was a statistically significant difference between the numbers of the various intestinal parasite species among the food handlers (p < 0.010). The parasite score density in the faecal specimens observed ranged between 1.50 for *Trichuris trichiura *whilst highest parasite score density was found among multiple infections with *Entamoeba histolytica *and *Ascaris lumbricoides *with 2.63.

**Table 3:** Distribution of intestinal parasites among examined food handlers.

Parasite class/species	Number of infected cases (n)	Infection rates (%)	% Of those examined. N = 168	Score of parasite density

Single Infections				
Protozoa				
*Entamoeba histolytica*	20	74.1	11.9	2.50
*Giardia lamblia*	6	22.2	3.6	1.67
*Balantidium coli*	1	3.70	0.6	2.00
Sub Total protozoa	27	100	16.1	
Helminthes				
*Ascaris lumbricoides*	22	59.5	13.1	2.55
Hookworms	13	35.1	7.7	2.38
*Trichuris trichura*	2	5.4	1.2	1.50
Sub Total Helminthes	37	100	22.0	
Multiple infections				
*E. histolytica+ A. lumbricoides*	3	60.0	1.8	2.63
*E. histolytica+G. lamblia*	2	40.0	1.2	2.56
Sub -total mixed infections	5	100	3.0	
Overall total of individuals	69		41.1	

## Discussion

The study showed high intestinal parasitic infestation of both meat and vegetable foodstuffs as 65.5 % and 75.9 %, respectively. Additionally, the burden of infection with intestinal parasites among the food handlers was almost half (41%). The presence of moderate parasite score densities in the foodstuffs and food handlers indicate high transmission risks of such parasites to inhabitants of Kisii Municipality. These findings indicate a public health priority and strongly support the need for education on food safety, Good Manufacturing Practice (GMP), Hazard Analysis Critical Control Pint (HACCP), improvement of sanitation conditions in and around the market by waste collection, management and handling of foodstuffs so as to reduce the prevalence of infections in both food handlers and food stuffs sold at the market.

Examination of vegetable samples revealed that all were equally highly contaminated with infestation rates of 52.4%, 57.1%, 81.0% and 71.4% respectively for kales, cabbage, spider flower, and black nightshade. This may be attributed to handling techniques of the vegetables [9]. The intestinal parasites found on the vegetable samples include protozoa: *Entamoeba histolytica, Giardia lamblia *and *Balantidium coli *and helminthes: *Ascaris lumbricoides*, *Trichuris trichiura *and hookworms. The risk of infection with intestinal parasites to the population is increased because these contaminated vegetables are sometimes eaten raw, undercooked to retain the natural taste and preserve heat-labile nutrients, or unclean [22]. Additionally, vegetables purchased at urban markets have been found to have higher rates of infestation with intestinal parasites [23].

The type of meat storage practice influenced the prevalence of intestinal parasites, with the refrigerator having least number (7%) while both meat placed in wire mesh and on open surface had higher number of intestinal parasites, 31.4% and 37.5% respectively. This indicates that proper storage methods like refrigeration should be used to minimize risk of contamination as only 18.1% of the butcheries sampled used refrigeration. Poor storage methods expose meat to mechanical vectors like houseflies, cockroaches and rats that transfer eggs, cysts of intestinal parasites to improperly stored meat [13]. Additionally, strong winds may blow eggs of intestinal parasites in dust to the exposed meat [24]. The butcheries where the handlers handled money while serving meat had 59.8% parasites prevalence while those where different personnel handled money and the meat serving had low prevalence (16.1%), this is major factor enhancing the risk of acquiring intestinal parasites as shown in other studies [11,25]. Besides, the cysts of intestinal parasites have been found to adhere on meat carcasses [26], this enhances the risk of transmission of intestinal parasites especially where meat is undercooked or eaten raw.

The most common intestinal parasites affecting the food handlers were *A. lumbricoides *(13.1%) and *E. histolytica *(11.9%), as was observed in Kisii District [16] although the study did not show intestinal protozoans. This contrasts sharply with earlier studies done in poor periurban and urban communities in Nairobi that had *A. lumbricoides *prevalence of 82% [27]. The prevalence of infection with intestinal parasites among the food handlers was almost half (41.1%) of those examined, with 3% having multiple protozoan and helminth infections. This high prevalence is a risk to patrons especially if the handlers fail to adequately sanitize hands, use food handling tools (tongs, spoons, utensils or bakery or serving papers) and handle money while serving food [28]. Foods sold in markets may be contaminated from hands that have not been washed after defeacation or from flies that land on both food and faeces hence increasing risks of transmission of intestinal parasites to consumers [29].

## Conclusion

About half of the people surveyed (41.1 %) at the Municipal Hospital had one or more parasitic infections. Furthermore, meat (65.5%) and vegetables (75.9%) sold at the Municipal market were found to be contaminated with parasites. The handlers of meat should employ proper storage methods like refrigeration, employ different personnel to handle money and serve meat to consumers. Additionally, they should use insecticides to kill the houseflies. Media programmes should be launched to enlighten the public on the necessity of good sanitation hygiene and risks of acquiring intestinal parasites.

## Competing interests

The authors declare that they have no competing interests.

## Authors' contributions

RMN designed, performed sampling, parasitological examination and manuscript development. PAA Contributed in planning the research, discussing the results and revising the manuscript. EWK participated in the study design, coordinated the study and revised the manuscript. BON conducted statistical analysis, discussing the results and developing the manuscript. All authors read and approved the final manuscript.

## Pre-publication history

The pre-publication history for this paper can be accessed here:


